# Clinical experience with an active intravascular rewarming technique for near-severe hypothermia associated with traumatic injury

**DOI:** 10.1186/2052-0492-2-11

**Published:** 2014-02-18

**Authors:** Kazutaka Kiridume, Toru Hifumi, Kenya Kawakita, Tomoya Okazaki, Hideyuki Hamaya, Natsuyo Shinohara, Yuko Abe, Koshiro Takano, Masanobu Hagiike, Yasuhiro Kuroda

**Affiliations:** Emergency Medical Center, Kagawa University Hospital, 1750-1 Ikenobe, Miki, Kita, Kagawa, 761-0793 Japan

**Keywords:** Intravascular rewarming, Hypothermia, Coagulopathy, Trauma

## Abstract

Hypothermia and acidosis are secondary causes of trauma-related coagulopathy. Here we report the case of a 72-year-old patient with severe trauma who suffered near-severe hypothermia despite the initiation of standard warming measures and was successfully managed with active intravascular rewarming. The patient was involved in a road traffic accident and was transported to a hospital. He was diagnosed with massive right-sided hemothorax, blunt aortic injury, burst fractures of the eighth and ninth thoracic vertebrae, and open fracture of the right tibia. He was referred to our hospital, where emergency surgery was performed to control bleeding from the right hemothorax. During surgery, the patient demonstrated progressive heat loss despite standard rewarming measures, and his temperature decreased to 32.4°C. Severe acidosis was also observed. A Cool Line® catheter was inserted into the right femoral vein and lodged in the inferior vena cava, and an intravascular balloon catheter system was utilized for aggressive rewarming. The automated target core temperature was set at 37°C, and the maximum flow rate was used. His core temperature reached 36.0°C after 125 min of intravascular rewarming. The severe acidosis was also resolved. The main active bleeding site was not identified, and coagulation hemostasis as well as rewarming enabled us to control bleeding from the vertebral bodies, lung parenchyma, and pleura. The total volume of intraoperative bleeding was 5,150 mL, and 20 units of red cell concentrate and 16 units of fresh frozen plasma were transfused. After surgery, he was transferred to the intensive care unit under endotracheal intubation and mechanical ventilation. His hemodynamic condition stabilized after surgery. The rewarming catheter was removed on day 2 of admission, and no bleeding, infection, or thrombosis associated with catheter placement was observed. Extubation was performed on day 40, and his subsequent clinical course was uneventful. He recovered well following rehabilitation and was discharged on day 46. These findings suggest that active intravascular rewarming should be considered as an aggressive, additional rewarming technique in patients with near-severe hypothermia associated with traumatic injury.

## Background

Hypothermia, acidosis, and hemodilution are the three main secondary causes of trauma-related coagulopathy [1]. Although mild isolated hypothermia (defined as 33°C–35°C) does not have severe effects on hemostasis in the usual clinical setting of trauma [1], severe hypothermia with a core body temperature of 32°C primarily slows the onset of thrombin generation, thus interfering with hemostatic processes directly [2].

Internal rewarming devices that use countercurrent heat exchange are frequently used as effective methods to rewarm the critically injured patient in clinical practice [3–5]; however, their utility is limited when patients have ongoing heat loss from open body cavities during emergency surgery [6].

The intravascular balloon catheter system has been approved in the USA for therapeutic human core cooling and rewarming during or after cardiac or neurological surgery and after stroke [7]. Only a limited number of cases of severe hypothermia associated with traumatic injury managed by this active intravascular rewarming technique are published [6], and none have been published in Japan because this technique is not approved for use in trauma management.

Here we report the case of a 72-year-old patient with severe trauma who developed near-severe hypothermia despite the initiation of standard warming measures, including convective heated air blankets, intravenous fluids, and a blood product inline warming machine. The hypothermia was successfully controlled by active intravascular rewarming using this closed-circuit, thermostatically controlled, warm water-circulating balloon catheter.

## Case presentation

**Figure 1:**
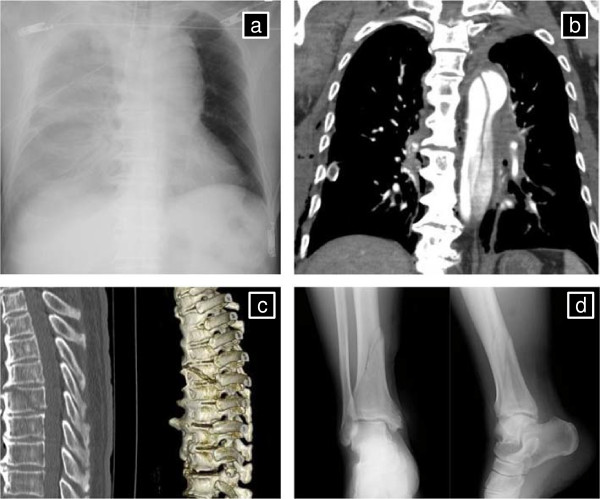
**Details of injuries. (a)** Chest X-ray showing massive right hemothorax. **(b)** CT reconstruction showing traumatic aortic dissection. **(c)** CT reconstruction showing fracture of the eighth and ninth thoracic vertebrae. **(d)** X-ray showing fracture of the right tibia.

A 72-year-old man was involved in a road traffic accident and was admitted to a local hospital in 30 min; he was diagnosed with massive right hemothorax, blunt aortic injury, fracture of the eighth and ninth thoracic vertebrae, and an open fracture of the right tibia (Figure 1). Head computed tomography (CT) showed no abnormalities. Abbreviated Injury Scale scores were as follows: head/neck 2, face 1, thorax 4, abdomen 4, extremities 2, external 1, and injury severity score 36.

Tracheal intubation and chest drainage of right hemithorax were required to treat hypovolemic shock caused by massive hemothorax, and following prompt wound cleansing and irrigation of the right tibia, he was transferred to our hospital for further treatment.

**Figure 2:**
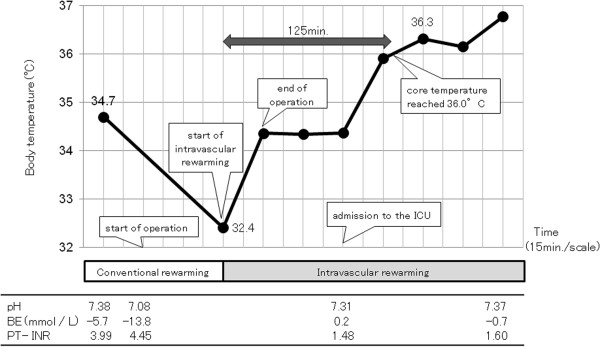
**Hospital course and body temperature.** The patient demonstrated progressive heat loss despite standard rewarming measures, and his temperature fell to 32.4°C. Also, severe acidosis (pH 7.08, base excess (BE) –13.8 mmol/L) was observed. The intravascular balloon catheter system was used for aggressive rewarming. His core temperature reached 36.0°C after 125 min of intravascular rewarming, and the severe acidosis was normalized.

The hospitalization course is shown in Figure 2.

On initial examination (190 min after initial injury), the patient's level of consciousness decreased, with a Glasgow Coma Scale score of 10/15. His vital signs were as follows: body temperature 34.7°C, blood pressure 50/30 mmHg, heart rate 96 beats/min, and respiratory rate 12 breaths/min. His peripheral arterial oxygen saturation was 100% with mechanical ventilation (controlled mandatory ventilation; F_I_O_2_ 1.0; tidal volume 500 mL).

**Figure 3:**
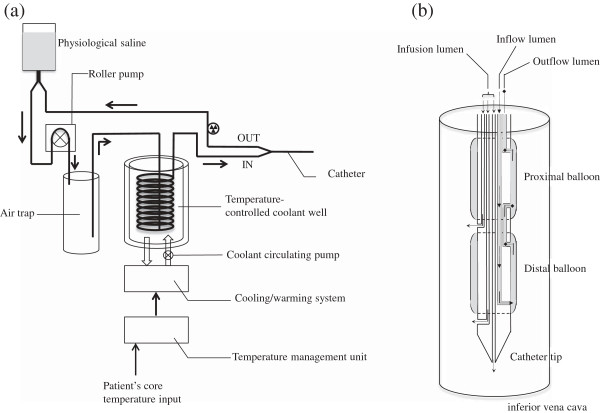
**Thermogard and rewarming catheter. (a)** The Thermogard XP® system, which remotely senses changes in the patient's core temperature, automatically adjusts this to the target set by the use of a catheter incorporating circulating saline (reprinted courtesy of AsahiKASEI ZOLL Medical). The machine acts as a thermostat for core body temperature control, with a user-selected target temperature (31°C–38°C). Sterile saline from a standard 500-mL hanging bag is actively pumped through the machine and the intravascular catheter balloons in a closed loop at 200–240 mL/min, depending on the catheter type. Within the machine, the saline passes first through an air trap, followed by passing through a metal heat exchanger coil submerged in a temperature-controlled coolant well containing a mixture of propylene glycol and distilled water. The saline then circulates through balloons on the intravascular surface of one of the specially designed central venous catheters at a temperature of 0°C to 42°C to deliver or remove heat from the bloodstream. **(b)** The Cool Line® catheter is inserted into the common femoral vein and lodges in the inferior vena cava. Saline flow within the balloon creates a proprietary vortex flow pattern, which maximizes heat exchange with blood as it passes through (reprinted courtesy of AsahiKASEI ZOLL Medical).

Twenty minutes after arrival, he was transferred to the operating room for the control of bleeding from the right hemithorax. Conservative treatment for the aortic injury included strict blood pressure control, considering the risk of open surgical repair and unavailability of emergency endovascular aortic repair. Exploratory thoracotomy in the right lateral position was performed because of the initial 1,500 mL of blood drainage followed by the persistent bleeding from the right hemithorax. During surgery, the patient demonstrated progressive heat loss despite standard rewarming measures, and his temperature decreased to 32.4°C. Severe acidosis was also observed (pH 7.08, base excess (BE) –13.8 mmol/L). A Cool Line® catheter (AsahiKASEI ZOLL Medical, Tokyo, Japan) was inserted into the right femoral vein and lodged in the inferior vena cava, and the intravascular balloon catheter system (Thermogard XP® system, AsahiKASEI ZOLL Medical) was used for aggressive rewarming (Figure 3). The automated target core temperature was set at 37°C, and maximum flow rate was used. His core temperature reached 36.0°C after 125 min of intravascular rewarming at an average warming rate of 2.2°C/h. The severe acidosis also normalized (pH 7.31; BE 0.2 mmol/L). Institutional review board approval was obtained for the use of the intravascular balloon catheter system. Written informed consent was obtained from the patient's family.

Although the main active bleeding site was not identified, coagulation hemostasis and rewarming enabled us to control bleeding from vertebral bodies, lung parenchyma, and pleura. The total volume of intraoperative bleeding was 5,150 mL, and 20 units of red cell concentrate and 16 units of fresh frozen plasma were transfused. The surgical duration was 94 min.

After surgery, he was transferred to the intensive care unit (ICU) under endotracheal intubation and mechanical ventilation. His hemodynamic condition gradually stabilized, and the rewarming catheter was removed on day 2 after admission. No bleeding, infection, or thrombosis associated with catheter placement was observed. Tracheostomy was performed for expected prolonged mechanical ventilation on day 5, and the patient was transferred to a general ward on day 22. He was extubated on day 40, and his subsequent clinical course was uneventful. He recovered well following rehabilitation and was discharged on day 46. With regard to the vertebral fractures, we provided precise information on the risks and benefits of surgery to the patient's family, who chose conservative treatment. No apparent aortic aneurysm or progressive aortic dissection was observed during 1 year of outpatient follow-up.

Here we reported the case of a patient with serious multiple trauma whose condition deteriorated because of near-severe hypothermia despite the initiation of standard warming measures, including convective heated air blankets, intravenous fluids, and blood product inline warming machines. The near-severe hypothermia was controlled successfully with an active intravascular rewarming technique using a closed-circuit, thermostatically controlled, warm water-circulating balloon catheter.

In patients undergoing emergency surgery for trauma, external rewarming modalities such as blanket-type rewarming and arctic sun® are inappropriate because a large part of the body's surface area needs to rewarm effectively. Several previous studies on critically injured patients have analyzed the multifactorial advantages of rapid internal rewarming techniques such as those that use countercurrent heat exchange and extracorporeal blood rewarming. These studies concluded that extracorporeal blood rewarming is currently the most effective method in regard to the average core rewarming rate [8–10]. However, the extracorporeal rewarming technique is invasive, requiring the placement of two large-bore catheters as well as adequate systematic blood pressure monitoring to ensure shunting of blood through the warming device [11, 12]. Furthermore, its operation requires the presence of a trained nurse or technician.

The most frequently used rewarming devices involving countercurrent heat exchange do not require large-bore catheter placement and are not vulnerable to the formation of air emboli [3]; however, such methods require continuous administration of warmed fluids to maintain core temperature. Furthermore, excessive administration of fluids without the inclusion of fresh frozen plasma (FFP) in the resuscitation phase following trauma may induce hemodilution of coagulation factors [13]. Both hypothermia and hemodilution cause trauma-induced secondary coagulopathy. Hemodilution can be prevented by the administration of adequate volumes of FFP by this method [14, 15], but maintaining core temperature can be problematic when aggressive fluid resuscitation is not required.

The rewarming method employed for the patient described above has several potential advantages. First, it does not require large-bore catheter placement. Second, it does not put the patient at risk of hypotensive events caused by extracorporeal circulation. Third, it can control core temperature without aggressive fluid administration. In patients with severe head injury, initial rewarming followed by therapeutic hypothermia can be initiated in the ICU using this method [16]. The risk of hemorrhage or air embolus is also minimal because there is no direct contact with the intravascular circulation [6].

This method requires the placement of a rewarming balloon catheter; however, because central venous access catheters are placed early in the resuscitation procedure in most patients with severe injuries, a rewarming balloon catheter is simply another type of central venous catheter.

In the present patient, no bleeding, infection, or thrombosis associated with catheter placement was observed. Although the catheter used in this system has warming balloons on the surface of the intravascular portion, the incidence of complications does not seem to be different from that associated with the use of central venous catheters.

## Conclusions

In conclusion, active intravascular rewarming should be considered as an aggressive, additional rewarming technique in patients with near-severe hypothermia associated with traumatic injury.

## Consent

Written informed consent was obtained from the patient for publication of this case report and any accompanying images, a copy of which is available for review by the Editor-in-Chief of this journal.
